# Supplementary material for: Fast mode decomposition in few-mode fibers
Source: Nat Commun. 2020 Nov 2;11:5507. doi: 10.1038/s41467-020-19323-6 (PMC7606473; doi:10.1038/s41467-020-19323-6)
Supplement: Supplementary file 1 — Supplementary Information [file 41467_2020_19323_MOESM1_ESM.pdf]

Supplementary Materials

Egor S. Manuylovich et al.

Supplementary Note 1: On the properties of the matrix of pairwise products of eigenmodes

In order to understand the limits of the method in the mode decomposition problem, we need to study in more detail the properties of the matrix of pairwise products of eigenmodes **T** (see eq. 7 in the article):

$$\mathrm{T}=\begin{pmatrix} \Psi_1^{(1)}\Psi_1^{(1)} & \cdots & \Psi_N^{(1)}\Psi_N^{(1)} & 2\Psi_1^{(1)}\Psi_2^{(1)} & \cdots & 2\Psi_1^{(1)}\Psi_N^{(1)} & 2\Psi_2^{(1)}\Psi_3^{(1)} & \cdots & 2\Psi_2^{(1)}\Psi_N^{(1)} & \cdots & 2\Psi_{N-1}^{(1)}\Psi_N^{(1)} \\ \vdots & & \vdots & \vdots & & \vdots & \vdots & & \vdots & & \vdots \\ \Psi_1^{(m)}\Psi_1^{(m)} & \cdots & \Psi_N^{(m)}\Psi_N^{(m)} & 2\Psi_1^{(m)}\Psi_2^{(m)} & \cdots & 2\Psi_1^{(m)}\Psi_N^{(m)} & 2\Psi_2^{(m)}\Psi_3^{(m)} & \cdots & 2\Psi_2^{(m)}\Psi_N^{(m)} & \cdots & 2\Psi_{N-1}^{(m)}\Psi_N^{(m)} \\ \vdots & & \vdots & \vdots & & \vdots & \vdots & & \vdots & & \vdots \\ \Psi_1^{(M^2)}\Psi_1^{(M^2)} & \cdots & \Psi_N^{(M^2)}\Psi_N^{(M^2)} & 2\Psi_1^{(M^2)}\Psi_2^{(M^2)} & \cdots & 2\Psi_1^{(M^2)}\Psi_N^{(M^2)} & 2\Psi_2^{(M^2)}\Psi_3^{(M^2)} & \cdots & 2\Psi_2^{(M^2)}\Psi_N^{(M^2)} & \cdots & 2\Psi_{N-1}^{(M^2)}\Psi_N^{(M^2)} \end{pmatrix}$$

The number of columns in the matrix **T** equals to the length of vector **z** and equals to $N_z = N_m\cdot(N_m+1)/2$. The matrix can we written in the form:

$$\mathbf{T}=\begin{pmatrix} t_1^{(1)} & \cdots & t_{N_z}^{(1)} \\ \vdots & & \vdots \\ t_1^{(m)} & \cdots & t_{N_z}^{(m)} \\ \vdots & & \vdots \\ t_1^{(M^2)} & \cdots & t_{N_z}^{(M^2)} \end{pmatrix} \tag{2}$$

We investigated how the rank of the matrix and the condition number depend on the number of modes. We noticed that with the number of modes $N = 10$, there is a sharp jump in condition number to about the inverse accuracy of double numbers:

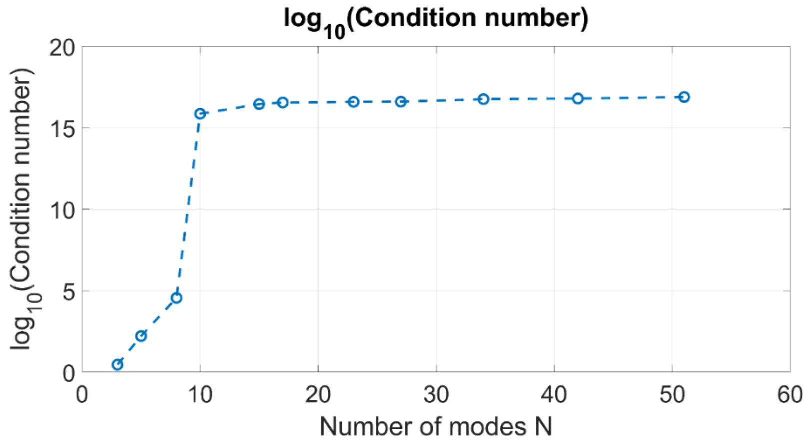

Supplementary Figure 1. Condition number of the matrix **T** depending on number of modes.

This means that with the number of modes $N = 10$, the matrix columns become linearly dependent. We calculated the angles between all pairs of columns and found that there are 2 types of anomalies: exactly collinear pairs of

columns and approximately collinear (with an accuracy of 10^{-3}-10^{-2}) pairs of columns. These second anomalous pairs of columns aroused the greatest interest, since one column from such a pair always contains the product of the fundamental mode LP_{01} and some other higher mode. This is especially important, because we directly calculate the phase difference between this higher mode and the fundamental mode using these pairwise products.

From a certain number of modes there are exactly coinciding columns $t = \Psi_j \Psi_k$ that correspond to pairwise products of sin- and cos- submodes, for example for LP_{11} и LP_{12} modes:

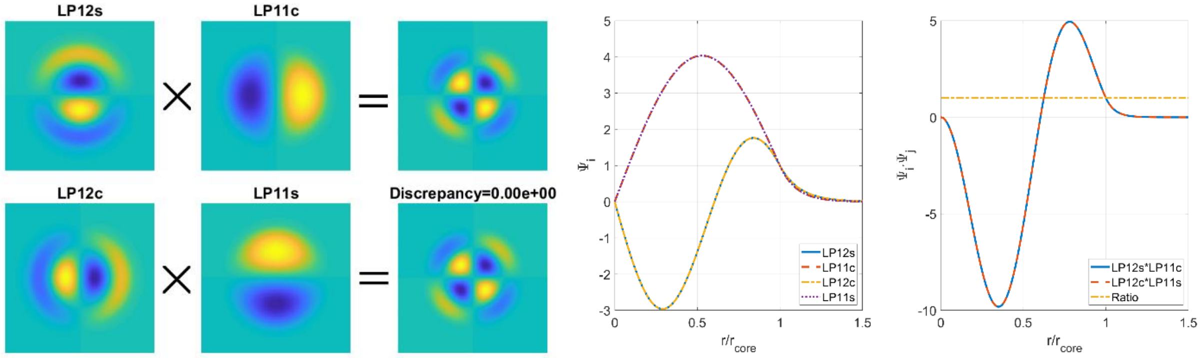

Supplementary Figure 2. Modes that form coincide columns in matrix **T**.

It is worth mentioning here that for each mode with $L > 0$ there are 2 orthogonal “submodes” $^{sin}LP_{11}$ and $^{cos}LP_{11}$, which we consider as separate modes. This leads to the appearance of coinciding columns in the matrix **T**.

In addition, there are very similar columns:

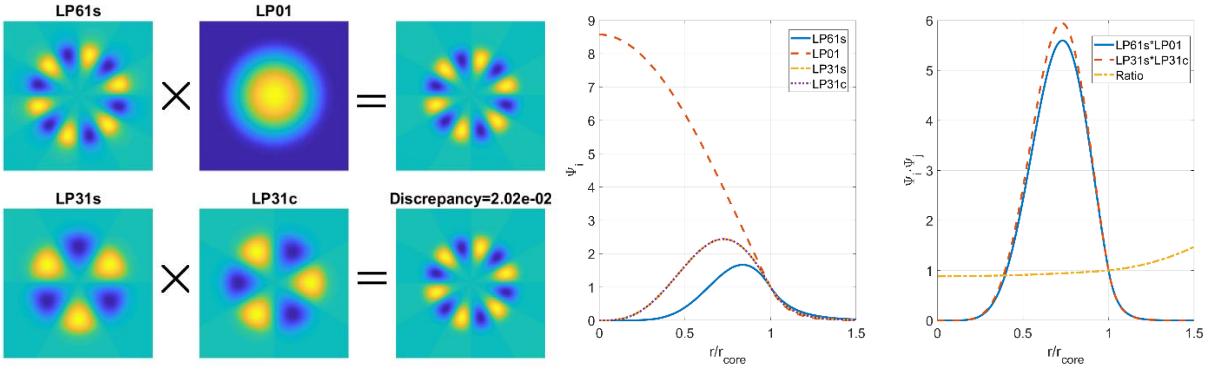

Supplementary Figure 3. Modes that form close columns in matrix **T**.

It should be noted that for “almost coincident” (in the picture above) the discrepancies are due to the difference in the transverse wave numbers for the modes LP_{31}, LP_{01} и LP_{61}.

Such coinciding columns result in the matrix being ill-conditioned, and it becomes impossible to take the pseudo-inverse matrix. Table 1 shows the matrix rank values depending on the number of modes. The value $N_z = N_m \cdot (N_m+1)/2$ is the number of columns in the matrix **T**. It should be noted that the linear independence of the columns can only be

determined with an accuracy of $\approx 2\cdot 10^{-16}$, which is determined by the accuracy of the double class. The fluctuations of the matrix rank are associated with this for a large number of modes with a sufficiently large size of the input image *M*.

$\operatorname{rank}(\mathbf{T})$, linearly independence accuracy = $2.22\cdot 10^{-16}$								
N	N_z	*M* = 8	*M* = 16	*M* = 32	*M* = 64	*M* = 128	*M* = 256	*M* = 512
3	6	6	6	6	6	6	6	6
5	15	15	15	15	15	15	15	15
8	36	36	36	36	36	36	36	36
10	55	51	54	54	54	54	54	54
15	120	64	118	118	118	118	118	118
17	153	64	151	151	151	151	151	151
23	276	64	248	271	271	271	271	271
27	378	64	256	372	372	371	370	367
34	595	64	256	517	520	512	509	505
42	903	64	256	640	658	654	645	638
51	1326	64	256	734	802	794	784	774

Supplementary Table 1. Rank of matrix $\mathbf{T}$ depending on number of modes and image size.

Starting from the number of modes *N* = 10, the rank of the matrix **T** does not coincide with the number of columns, since for the first time a combination of the LP_{11} and LP_{12} modes appears, which give the same columns for sin- and cos- components (see Fig. SP2). However, so far this has little effect on the accuracy of the decomposition, since many other linearly independent columns remain, which include coefficients for these modes.

There is a way to get rid of the ill conditioning of the matrix **T**. The first idea is to leave only one of the two columns, add the corresponding components of the vector **z** and renumber it.

For example, for the number of modes *N* = 10, the columns that correspond to the modes ${}^{\sin}LP_{11}\cdot{}^{\cos}LP_{12}$ and ${}^{\cos}LP_{11}\cdot{}^{\sin}LP_{12}$ coincide (see Supplementary Figure 2a).

Denote

$$t_p = {}^{\sin}\mathrm{LP}_{11}\cdot{}^{\cos}\mathrm{LP}_{12}$$
$$t_q = {}^{\cos}\mathrm{LP}_{11}\cdot{}^{\sin}\mathrm{LP}_{12}$$

Then change the variables $z_p' = z_p + z_q$, and remove the column t_q from **T**. We do this for all pairs of modes that form coinciding columns.

After that, there are no collinear pairs of columns in the matrix **T**, and for a small number of modes the condition number of the matrix decreases drastically:

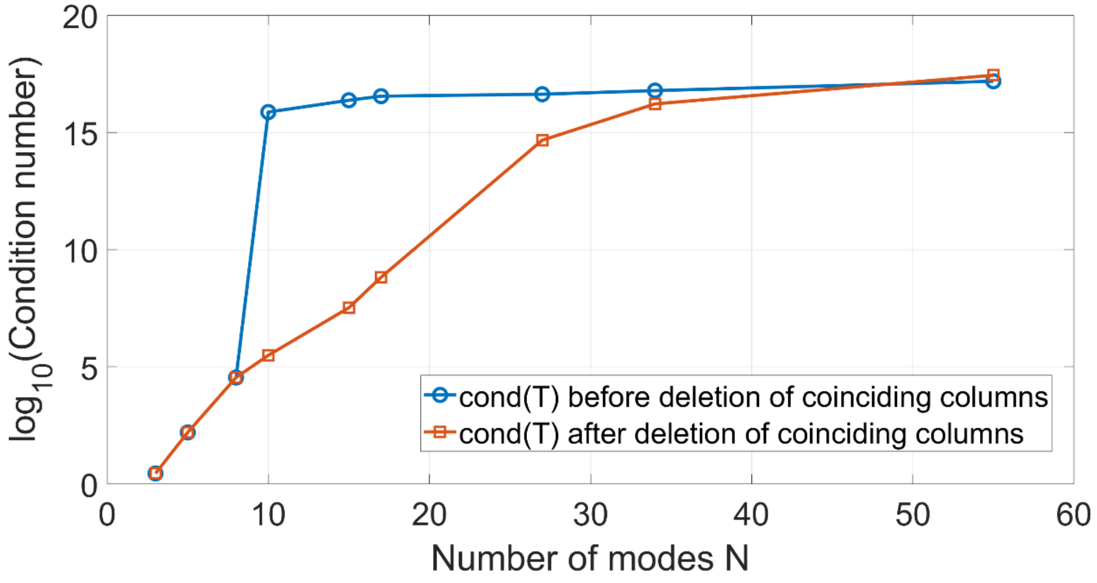

Supplementary Figure 4. Condition number of the matrix **T** before and after deletion of coinciding columns.

However, for number of modes $N \approx 30$ the condition number is almost the same even after deletion of coinciding columns. This means that there are coplanar columns and new columns appearing with the addition of new modes can be described by a linear combination of existing ones. For a coplanar set of vectors there is no universal way to make the change of variables in the vector **z** and renumber it.

It should be noted that this kind of symmetry can be eliminated if we perform an azimuthal rotation for each pair of sin- and cos-modes:

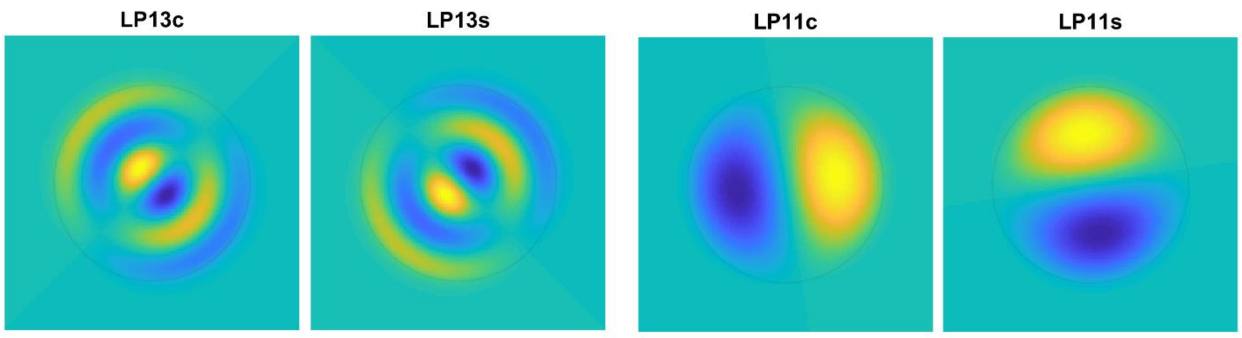

Supplementary Figure 5. Rotation of each pair of sub-modes at certain angle eliminates coinciding columns in matrix **T**.

But this trick doesn't lead neither to decreasing of condition number nor to increasing rank of the matrix **T**. It is another evidence for linear dependence of multiple columns in the matrix of pairwise products of eigenmodes which limits the maximum number of modes in a mode decomposition problem.

Supplementary Note 2: On error distribution

Non-symmetrical error distribution is observed for non-negative noise model. The thing is we can't use gaussian noise directly because when added to low-intensity pixels it can produce negative values of intensity.

That's why we use max[0, x] for each intensity distribution after adding gaussian noise. And it's something what is truly happening in an experiment. We found that the error distribution for decomposed weights is shifted:

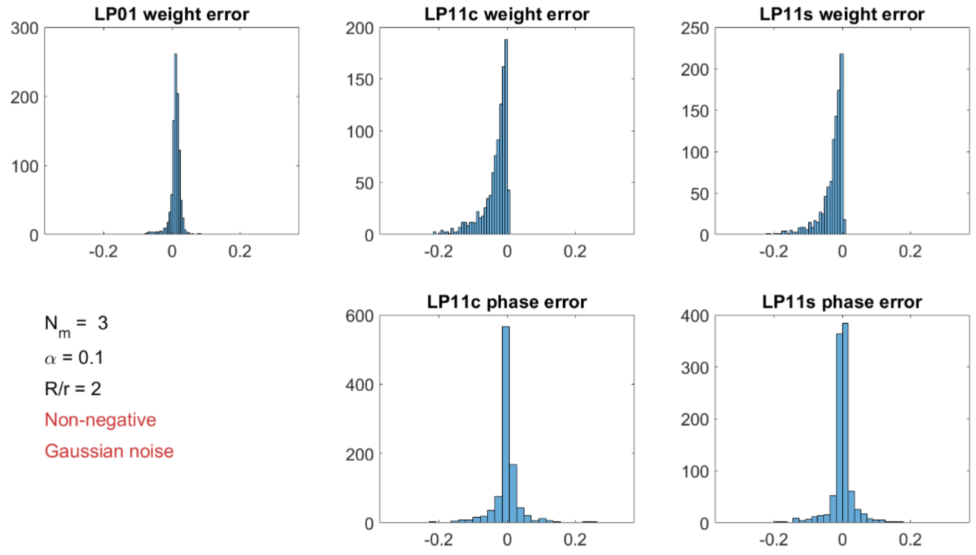

Supplementary Figure 6. Non-symmetrical error distribution for non-negative noise model

Compared to "true" gaussian noise (which can't be observed since there are some pixels with negative intensity):

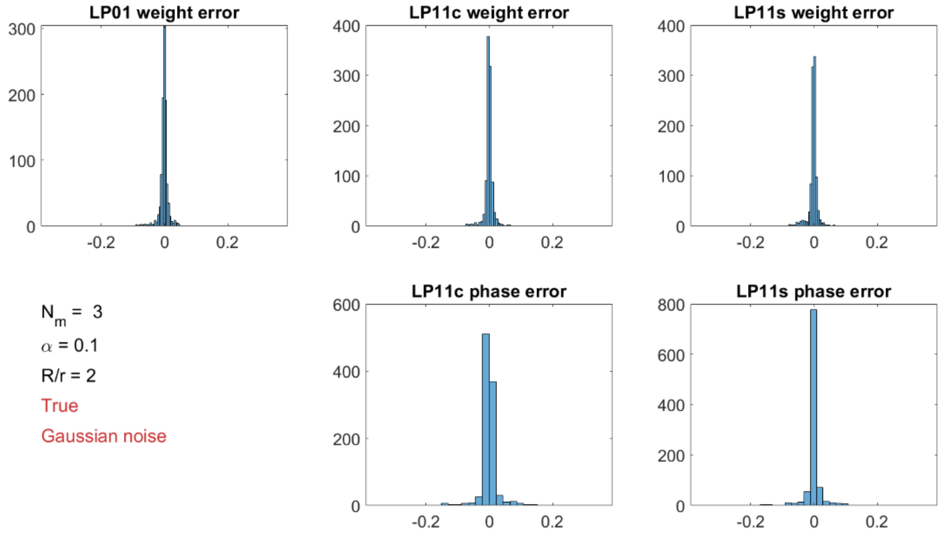

Supplementary Figure 7. For symmetrical noise distribution the decomposition error is also symmetrical.

This induces additional errors in mode weight distribution. We believe that using general message passing algorithm can help to get rid of this because it allows to consider noise model that is different from standard additive Gaussian white noise.
